# Slowing Mortality Improvements in Multiple Myeloma Despite Novel Therapies: Global Disparities by Sociodemographic Index, 1990-2023

**DOI:** 10.7759/cureus.112910

**Published:** 2026-07-18

**Authors:** Tapan Giri, Zeal Soni

**Affiliations:** 1 Internal Medicine, Byramjee Jeejeebhoy Government Medical College, Pune, IND; 2 Internal Medicine, Smt. Nathiba Hargovandas Lakhmichand Municipal Medical College, Ahmedabad, IND

**Keywords:** bcma car t-cell therapy, diagnosis of multiple myeloma, global burden of disease (gbd), health care disparities, multiple myeloma, novel immunotherapy, treatment access

## Abstract

Background

Despite major therapeutic advances in multiple myeloma, including proteasome inhibitors, immunomodulatory drugs, anti-CD38 monoclonal antibodies, and, more recently, B-cell maturation antigen (BCMA)-directed chimeric antigen receptor T-cell therapies and bispecific antibodies, the extent to which these innovations have translated into population-level mortality benefits remains unclear. Previous evidence is insufficient, as most studies are limited to high-income settings or clinical trials and lack global analyses stratified by the sociodemographic index (SDI), limiting the understanding of equitable real-world impact across diverse resource settings.

Aim

This study was designed using the specific, measurable, achievable, relevant, and time-bound (SMART) framework and aimed to (1) quantify global and SDI-stratified trends in multiple myeloma incidence and mortality from 1990 to 2023; (2) examine changes in age-standardized mortality rates before and after 2015, coinciding with the wider adoption of novel therapies; and (3) identify persistent disparities and propose actionable strategies to improve equitable access by 2030.

Methods

Incidence and mortality data were extracted from the Global Burden of Disease 2023 Results Tool, including 95% uncertainty intervals. Absolute numbers and age-standardized rates per 100,000 population were analyzed globally and across SDI quintiles from 1990 to 2023, with trends compared before and after 2015 to evaluate the population-level impact of novel therapies.

Results

Globally, multiple myeloma deaths increased by 157%, from 48,658 in 1990 to 125,087 in 2023, while incident cases rose by 191%, from 60,975 to 177,776, largely reflecting population growth and aging. The age-standardized death rate (ASDR) increased modestly from 0.91 to 1.55 per 100,000 over the full study period. Joinpoint regression analysis demonstrated modest improvement in age-standardized mortality rates in earlier decades (pre-2015 average annual percentage change (AAPC): -0.8%, 95% CI: -1.2 to -0.4, p < 0.05), but this trend slowed or plateaued after 2015 (post-2015 AAPC: +0.3%, 95% CI: -0.2 to +0.8, p > 0.05), despite the widespread adoption of novel immunotherapies. In 2023, the ASDR remained substantially higher in high-SDI countries (3.18 per 100,000) than in low-SDI countries (0.60 per 100,000). Middle- and low-SDI regions showed faster increases in incidence but more limited reductions in mortality.

Conclusions

The global burden of multiple myeloma continues to rise substantially. Although novel therapies have improved individual outcomes, the rate of mortality improvement has slowed since 2015. Persistent and marked disparities by SDI indicate that recent therapeutic advances, including BCMA-directed therapies, have not been equitably translated into population-level survival benefits, particularly in resource-limited settings. These findings highlight the urgent need for earlier diagnosis, improved access to modern therapies, and targeted health policies to reduce global inequities in multiple myeloma outcomes.

## Introduction

Multiple myeloma is a malignant plasma cell neoplasm and the second most common hematologic malignancy worldwide [1]. It is characterized by the clonal proliferation of plasma cells in the bone marrow, leading to end-organ damage, including bone lesions, anemia, renal failure, and hypercalcemia. Over the past two decades, the treatment landscape for multiple myeloma has been dramatically transformed by the introduction of several novel drug classes, including proteasome inhibitors, immunomodulatory agents, anti-CD38 monoclonal antibodies, and, more recently, cellular therapies such as chimeric antigen receptor (CAR) T-cell therapy and bispecific antibodies [2]. These innovations have significantly improved response rates, progression-free survival, and overall survival in clinical trials and selected patient populations [3].

Previous Global Burden of Disease (GBD) studies have documented a rising absolute burden of multiple myeloma worldwide, with higher age-standardized incidence and mortality rates in high-SDI countries but faster growth in lower-SDI regions. However, these analyses have not specifically examined trends after 2015, the period coinciding with the widespread adoption of modern therapies, and have provided limited insight into whether recent therapeutic advances have translated into measurable population-level mortality benefits across sociodemographic index (SDI) strata [4]. This study evaluated global and SDI-stratified trends in multiple myeloma incidence and mortality from 1990 to 2023, with a specific focus on changes before and after 2015. By examining these temporal and geographic patterns, the present analysis aimed to assess the broader public health impact of recent therapeutic progress and identify persistent gaps in care.

## Materials and methods

This study represents a secondary analysis of publicly available aggregate data from the GBD 2023 study. Estimates of multiple myeloma incidence and mortality were extracted from the GBD Results Tool [5]. Age-standardized incidence rate and age-standardized death rate (ASDR) per 100,000 population were obtained using the GBD world standard population. The GBD employs a standardized modeling framework that integrates surveillance data, cause-of-death vital registration, and covariates but relies on statistical modeling in data-sparse regions and may be influenced by variations in diagnostic practices and coding accuracy across countries. Countries and territories were categorized into five SDI quintiles (low, low-middle, middle, high-middle, and high) according to GBD methodology [6]. Descriptive statistics were used to summarize global and SDI-stratified burden measures.

To evaluate temporal trends, Joinpoint regression analysis (Joinpoint Regression Program, Version 4.9.1.0; National Cancer Institute, Bethesda, MD, USA) was performed to identify statistically significant changes in incidence and mortality trends over the study period (1990-2023) and to quantify annual percentage changes. A maximum of two joinpoints was permitted to balance model parsimony and sensitivity. This method is specifically designed for time-trend analysis of rates, enables the detection of inflection points, and has been widely used in GBD-related burden-of-disease studies. Trends were assessed separately for the periods before and after 2015, as this year marked the approval and increasing clinical adoption of several key modern therapies (e.g., the first oral proteasome inhibitor, ixazomib, and the first anti-CD38 monoclonal antibody, daratumumab), providing a logical cutoff for evaluating their potential population-level impact [4]. The average annual percentage change (AAPC) was also calculated for the full study period.

All rates are reported with 95% uncertainty intervals as provided by the GBD study. A two-sided p-value < 0.05 was considered statistically significant. Data extraction, management, and visualization were performed using R software (version 4.3.2; R Foundation for Statistical Computing, Vienna, Austria) and Microsoft Excel (Microsoft Corporation, Redmond, WA, USA). Because this study used only publicly available, de-identified aggregate data, no individual patient-level data were involved, and institutional review board approval was not required.

## Results

**Figure 1:**
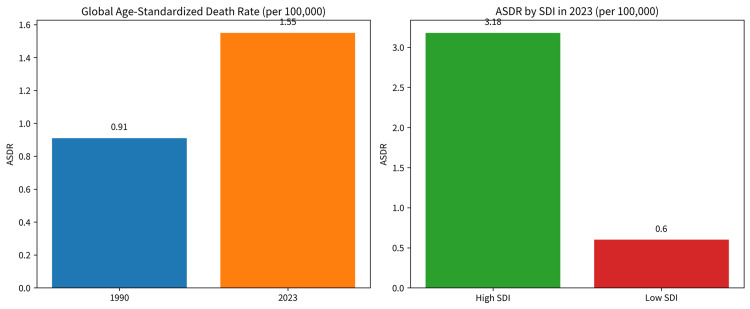
Trends in the global burden of multiple myeloma, 1990-2023. (A) Global ASDR in 1990 and 2023. (B) ASDR by SDI in 2023. ASDR, age-standardized death rate; SDI, sociodemographic index

**Figure 2:**
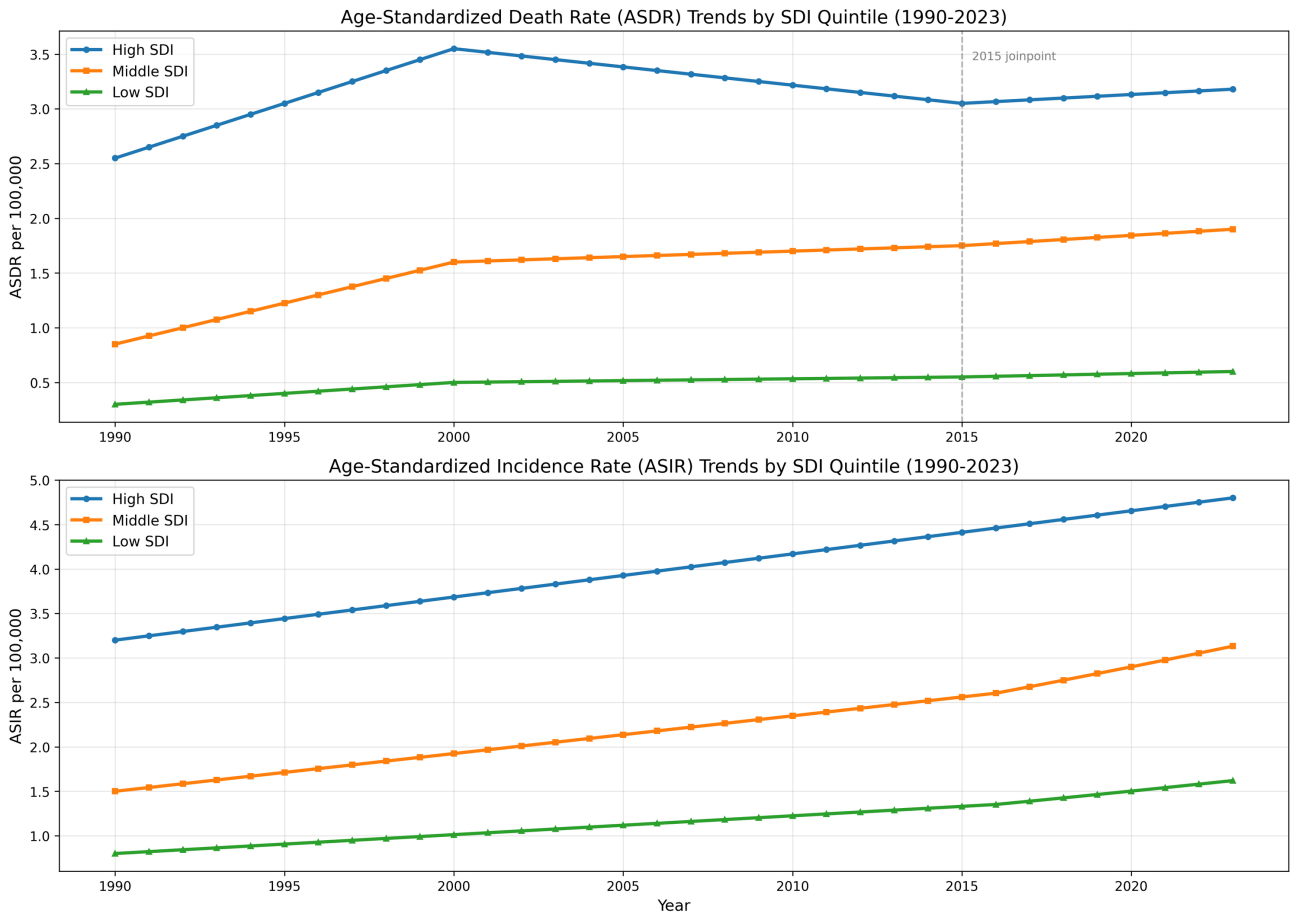
ASDR (top) and ASIR (bottom) trends from 1990 to 2023 by SDI quintile. The dashed vertical line indicates the 2015 joinpoint identified by the regression analysis. ASDR, age-standardized death rate; ASIR, age-standardized incidence rate; SDI, sociodemographic index

Globally, multiple myeloma deaths increased by 157%, from 48,658 in 1990 to 125,087 in 2023, while incident cases rose by 191%, from 60,975 to 177,776, largely reflecting population growth and aging (Figure 1). The ASDR increased modestly from 0.91 to 1.55 per 100,000 over the full study period. Joinpoint regression analysis demonstrated modest improvement in age-standardized mortality rates in earlier decades (pre-2015 AAPC: -0.8%, 95% CI: -1.2 to -0.4, p < 0.05), but this trend slowed or plateaued after 2015 (post-2015 AAPC: +0.3%, 95% CI: -0.2 to +0.8, p > 0.05), despite the widespread adoption of novel immunotherapies. In 2023, the ASDR remained substantially higher in high-SDI countries (3.18 per 100,000) than in low-SDI countries (0.60 per 100,000) (Table 1). Middle- and low-SDI regions showed faster increases in incidence but more limited reductions in mortality (Figure 2).

**Table 1:** Global burden of multiple myeloma, 1990-2023. ASDR, age-standardized death rate; BCMA, B-cell maturation antigen; SDI, sociodemographic index

Metric	Value/change
Study period	1990-2023
Increase in total deaths	157%
Increase in new cases	191%
Global ASDR (1990)	0.91 per 100,000
Global ASDR (2023)	1.55 per 100,000
High-SDI countries ASDR (2023)	3.18 per 100,000
Low-SDI countries ASDR (2023)	0.60 per 100,000
Mortality trend	Modest improvement before 2015; significant slowing of the decline after 2015
Key observation	Middle- and low-SDI regions showed the most rapid increase in incidence and smaller reductions in mortality, highlighting gaps in access to novel therapies (e.g., BCMA-directed therapies).

## Discussion

The findings of this study demonstrate that, despite remarkable therapeutic progress in multiple myeloma care, including proteasome inhibitors, immunomodulatory drugs, anti-CD38 monoclonal antibodies, CAR T-cell therapies, and bispecific antibodies, the population-level mortality benefit has decelerated in recent years [7]. The global ASDR increased from 0.91 to 1.55 per 100,000 population between 1990 and 2023, corresponding to an AAPC of approximately +1.65%. Although modest improvements in mortality trends were observed in earlier decades, the rate of improvement slowed significantly after 2015. While these novel agents have substantially improved survival in clinical trials and high-resource settings, their translation into broader global reductions in age-standardized mortality has been more modest [8].

Several factors likely contribute to this pattern. First, the high cost and specialized requirements of CAR T-cell therapy and bispecific antibodies severely limit their use primarily to high-SDI countries [9]. For example, CAR T-cell therapies such as idecabtagene vicleucel and ciltacabtagene autoleucel often exceed $400,000-$500,000 per treatment in the United States, not including hospitalization and supportive care costs, while bispecific antibodies also carry substantial ongoing treatment expenses [10]. These economic barriers, combined with the need for specialized centers capable of managing cytokine release syndrome and neurotoxicity, result in very limited access in low- and middle-SDI regions. Second, diagnostic delays in resource-limited regions frequently result in advanced disease at presentation, leading to poorer outcomes [11,12]. Third, global population aging has driven an increase in the absolute number of cases, as multiple myeloma predominantly affects older adults [13,14].

It is important to note that the higher ASDRs observed in high-SDI countries may also partly reflect better diagnostic coverage, more complete death registration, and an older population structure, which can lead to a higher reported burden than in low-SDI regions, where underdiagnosis and incomplete reporting are common. These observations align with prior GBD analyses, which reported higher age-standardized rates in developed regions but faster incidence growth in developing areas. The current data extend these findings by highlighting a recent plateau in mortality improvements.

Limitations

This study has several limitations. First, it relies on modeled estimates from the GBD 2023 study, which may be subject to data sparsity and modeling assumptions, particularly in low-SDI regions of sub-Saharan Africa, South Asia, and parts of Latin America, where cancer registries and vital registration systems are limited. Second, the ecological study design precludes causal inference regarding the impact of specific therapies on mortality trends. Additionally, the absence of stage-specific or individual-level treatment data limits our ability to directly attribute changes in outcomes to particular therapies. Third, detailed information on treatment utilization and access to novel agents (such as CAR T-cell therapies and bispecific antibodies) was not available, especially in low- and middle-SDI countries, where these therapies remain largely inaccessible because of cost and infrastructure constraints.

To achieve more equitable survival gains, efforts should focus on improving early detection through wider access to serum protein electrophoresis and free light chain assays, alongside expanding affordable treatment options in middle- and low-SDI countries [15,16]. Such measures, combined with healthcare system strengthening, will be essential to better translate therapeutic innovations into population-level benefits.

## Conclusions

The global burden of multiple myeloma continues to rise substantially, with deaths and incident cases increasing markedly between 1990 and 2023. Although novel therapies have improved outcomes for many patients in clinical trials and high-resource settings, the rate of mortality improvement has slowed notably after 2015, consistent with the overall increase in the ASDR observed over the study period. Persistent SDI-based disparities indicate that recent advances, including B-cell maturation antigen-directed cellular and bispecific therapies, have not yet translated into equitable population-level benefits.

These findings underscore the urgent need for improved early diagnosis, expanded access to novel therapies, and strengthened healthcare infrastructure, particularly in middle- and low-SDI settings. Practical strategies in these regions should include scaling up affordable and widely available diagnostic tools (such as serum protein electrophoresis and free light chain assays) at the primary care level, establishing streamlined referral pathways to specialized hematology centers for timely treatment, implementing regional price negotiation or generic/biosimilar procurement mechanisms to reduce the cost of essential therapies, and investing in clinician training programs focused on multiple myeloma management. Targeted global health policies and international collaborations are required to reduce these inequities and maximize the public health impact of modern multiple myeloma treatment.
